# Supplementary material for: Relationship between baseline platelet-to-red blood cell distribution width ratio and all-cause mortality in non-traumatic subarachnoid hemorrhage: A retrospective analysis of the MIMIC-IV database
Source: PLoS One. 2025 Aug 22;20(8):e0330825. doi: 10.1371/journal.pone.0330825 (PMC12373194; doi:10.1371/journal.pone.0330825)
Supplement: S1 Table — (DOCX) [file pone.0330825.s001.docx]

| **S1 Table.** Details of missing values. | | |
| --- | --- | --- |
| **Variables** | **The number of missing values** | **The percent of missing values** |
| Calciumtotal | 1 | <0.1% |
| Magnesium | 2 | 0.1% |
| PT | 25 | 2.3% |
| APTT | 28 | 2.6% |
| SBP | 20 | 1.8% |
| DBP | 20 | 1.8% |
| MAP | 20 | 1.8% |
| GCS | 1 | <0.1% |

Note: PT, Prothrombin Time; APTT, Activated Partial Thromboplastin Time; SBP, Systolic Blood Pressure; DBP, Diastolic Blood Pressure; MAP, mean arterial pressure; GCS, Glasgow Coma Scale.
