# Supplementary material for: Relationship between baseline platelet-to-red blood cell distribution width ratio and all-cause mortality in non-traumatic subarachnoid hemorrhage: A retrospective analysis of the MIMIC-IV database
Source: PLoS One. 2025 Aug 22;20(8):e0330825. doi: 10.1371/journal.pone.0330825 (PMC12373194; doi:10.1371/journal.pone.0330825)
Supplement: S2 Table — (DOCX) [file pone.0330825.s002.docx]

**S2 Table. Variance Inflation Factor and Tolerance**

| Term | ICU mortality | | In-hospital mortality | |
| --- | --- | --- | --- | --- |
|  | VIF | Tolerance | VIF | Tolerance |
| Gender | 1.536527 | 0.650818312 | 1.427104 | 0.700719689 |
| Race | 2.365386 | 0.422763895 | 2.029596 | 0.492708795 |
| Hypertension | 1.432434 | 0.698112418 | 1.439963 | 0.694462141 |
| Age | 2.283043 | 0.4380120 | 2.119726 | 0.4717592 |
| Diabetes | 1.695731 | 0.589716143 | 1.706443 | 0.586014380 |
| Heart failure | 1.839372 | 0.543663879 | 1.753142 | 0.570404365 |
| Myocardial infarction | 1.963212 | 0.509369336 | 1.835009 | 0.544956312 |
| Malignant tumor | 1.366298 | 0.731904572 | 1.222219 | 0.818183984 |
| Chronic kidney disease | 2.163563 | 0.462200506 | 2.000120 | 0.499970005 |
| Cirrhosis | 2.503507 | 0.399439718 | 2.168552 | 0.461137109 |
| Pneumonia | 1.440453 | 0.694226200 | 1.453815 | 0.687845371 |
| Hyperlipoidemia | 1.348539 | 0.741543447 | 1.246881 | 0.802001170 |
| Sepsis | 1.961714 | 0.509758304 | 1.854806 | 0.539139896 |
| Charlson | 2.675928 | 0.373702105 | 2.586723 | 0.386589466 |
| WBC | 1.679360 | 0.595464950 | 1.655348 | 0.604102727 |
| RBC | 10.788064 | 0.092695036 | 9.513165 | 0.105117482 |
| Platelet count | 2.237795 | 0.446868440 | 2.102796 | 0.475557248 |
| Hemoglobin | 11.269468 | 0.088735334 | 10.458109 | 0.095619577 |
| RDW | 2.709901 | 0.369017111 | 2.392885 | 0.417905615 |
| Sodium | 5.485718 | 0.182291527 | 4.511714 | 0.221645263 |
| Potassium | 2.628138 | 0.380497560 | 2.262782 | 0.441933819 |
| Magnesium | 1.691942 | 0.591036810 | 2.014803 | 0.496326552 |
| Calciumtotal | 2.321924 | 0.430677397 | 5.424522 | 0.184348046 |
| Chloride | 6.312372 | 0.158419061 | 1.841474 | 0.543043195 |
| Glucose | 1.813140 | 0.551529384 | 2.912777 | 0.343315033 |
| Aniongap | 3.476604 | 0.287637009 | 330.309633 | 0.003027462 |
| PT | 335.231286 | 0.002983015 | 1.442556 | 0.693214088 |
| APTT | 1.600283 | 0.624889636 | 334.623342 | 0.002988435 |
| INR | 339.091237 | 0.002949059 | 3.260740 | 0.306678829 |
| Ureanitrogen | 3.000060 | 0.333326645 | 2.453768 | 0.407536478 |
| Creatinine | 2.584507 | 0.386920925 | 1.144887 | 0.873448811 |
| Clipping | 1.138728 | 0.878173024 | 1.391610 | 0.718592361 |
| Coiling | 1.481927 | 0.674797067 | 1.447143 | 0.691016616 |
| Ventilation | 1.479912 | 0.675715672 | 1.677032 | 0.596291443 |
| HR | 1.710602 | 0.584589352 | 5.110447 | 0.195677604 |
| SBP | 8.444855 | 0.118415293 | 15.444101 | 0.064749642 |
| DBP | 39.184739 | 0.025520139 | 24.704031 | 0.040479223 |
| MBP | 62.020671 | 0.016123657 | 1.482235 | 0.674656653 |
| RR | 1.483955 | 0.673874665 | 1.366563 | 0.731762848 |
| SpO2 | 1.353299 | 0.738934779 | 1.667848 | 0.599574944 |
| Temperature | 1.529641 | 0.653748140 | 1.587428 | 0.629949789 |
| Dobutamine | 1.855976 | 0.538800033 | 1.752084 | 0.570748842 |
| Dopamine | 1.799186 | 0.555806983 | 1.834909 | 0.544986286 |
| Epinephrine | 2.137662 | 0.467800836 | 2.193704 | 0.455850113 |
| Norepinephrine | 2.132437 | 0.468947127 | 2.433540 | 0.410923930 |
| Vasopressin | 2.596180 | 0.385181334 | 5.145403 | 0.194348235 |
| SAPS Ⅱ | 5.950259 | 0.168059901 | 9.579483 | 0.104389768 |
| GCS | 11.004872 | 0.090868846 | 6.301759 | 0.158685849 |
| WFNS grade | 7.355099 | 0.135960102 | 1.532007 | 0.652738510 |
