# Supplementary material for: Relationship between baseline platelet-to-red blood cell distribution width ratio and all-cause mortality in non-traumatic subarachnoid hemorrhage: A retrospective analysis of the MIMIC-IV database
Source: PLoS One. 2025 Aug 22;20(8):e0330825. doi: 10.1371/journal.pone.0330825 (PMC12373194; doi:10.1371/journal.pone.0330825)
Supplement: S3 Table — (DOCX) [file pone.0330825.s003.docx]

**S3 Table. Patient demographics and baseline characteristics**

| **Characteristic** | **Is Hospital dead** | | | p-value |
| --- | --- | --- | --- | --- |
|  | Overall, N = 1,056 | Survival group, N = 860 | Non-survival group, N=196 |  |
| **Demographic** |  |  |  |  |
| Age, years | 61 (51, 72) | 60 (51, 71) | 68 (56, 79) | <0.001 |
| Gender, n (%) |  |  |  | 0.010 |
| Female | 599 (56.7%) | 504 (58.6%) | 95 (48.5%) |  |
| Male | 457 (43.3%) | 356 (41.4%) | 101 (51.5%) |  |
| **Race, n (%)** |  |  |  | <0.001 |
| White | 609 (57.7%) | 529 (61.5%) | 80 (40.8%) |  |
| Black | 74 (7.0%) | 63 (7.3%) | 11 (5.6%) |  |
| Asian | 43 (4.1%) | 34 (4.0%) | 9 (4.6%) |  |
| Other | 330 (31.3%) | 234 (27.2%) | 96 (49.0%) |  |
| **Comorbidities, n (%)** |  |  |  |  |
| Hypertension | 535 (50.7%) | 445 (51.7%) | 90 (45.9%) | 0.141 |
| Diabetes | 216 (20.5%) | 169 (19.7%) | 47 (24.0%) | 0.175 |
| Heart failure | 75 (7.1%) | 51 (5.9%) | 24 (12.2%) | 0.002 |
| Myocardial infarction | 35 (3.3%) | 18 (2.1%) | 17 (8.7%) | <0.001 |
| Malignant tumor | 94 (8.9%) | 80 (9.3%) | 14 (7.1%) | 0.338 |
| Chronic kidney disease | 64 (6.1%) | 42 (4.9%) | 22 (11.2%) | <0.001 |
| Cirrhosis | 25 (2.4%) | 10 (1.2%) | 15 (7.7%) | <0.001 |
| Pneumonia | 217 (20.5%) | 157 (18.3%) | 60 (30.6%) | <0.001 |
| Hyperlipoidemia | 289 (27.4%) | 230 (26.7%) | 59 (30.1%) | 0.341 |
| Sepsis | 510 (48.3%) | 372 (43.3%) | 138 (70.4%) | <0.001 |
| Charlson comorbidity index | 4.0 (2.0, 6.0) | 3.0 (2.0, 5.0) | 5.0 (3.0, 7.0) | <0.001 |
| **Vital signs** |  |  |  |  |
| HR, beats/min | 80 (70, 91) | 79 (69, 90) | 84 (71, 97) | 0.004 |
| SBP, mmHg | 128 (114, 142) | 129 (115, 143) | 128 (111, 141) | 0.153 |
| DBP, mmHg | 71 (62, 80) | 71 (62, 80) | 70 (59, 82) | 0.261 |
| MAP, mmHg | 86 (76, 95) | 86 (76, 95) | 86 (73, 97) | 0.484 |
| RR, times/min | 17.0 (15.0, 20.0) | 17.0 (15.0, 20.0) | 18.0 (16.0, 23.0) | <0.001 |
| SpO_2_, % | 98.0 (96.0, 100.0) | 98.0 (96.0, 100.0) | 99.0 (96.0, 100.0) | 0.005 |
| Temperature, ℃ | 36.83 (36.56, 37.11) | 36.89 (36.56, 37.11) | 36.78 (36.39, 37.13) | 0.006 |
| **Laboratory results** |  |  |  |  |
| WBC, K/uL | 11.2 (8.6, 14.5) | 10.8 (8.4, 13.9) | 12.9 (10.3, 17.3) | <0.001 |
| RBC, M/uL | 4.05 (3.66, 4.44) | 4.07 (3.69, 4.44) | 3.89 (3.37, 4.47) | 0.005 |
| Platelets, K/uL | 214 (172, 261) | 217 (178, 264) | 192 (140, 246) | <0.001 |
| Hemoglobin, g/dL | 12.30 (11.10, 13.43) | 12.30 (11.20, 13.50) | 11.90 (10.20, 13.30) | 0.009 |
| RDW, % | 13.40 (12.80, 14.30) | 13.30 (12.80, 14.00) | 14.05 (13.20, 15.20) | <0.001 |
| Sodium, mEq/L | 139.0 (137.0, 141.0) | 139.0 (137.0, 141.0) | 140.0 (137.0, 143.0) | 0.070 |
| Potassium, mEq/L | 3.90 (3.60, 4.20) | 3.80 (3.60, 4.20) | 4.00 (3.58, 4.50) | 0.002 |
| Magnesium, mg/dL | 1.90 (1.70, 2.10) | 1.90 (1.70, 2.00) | 1.90 (1.70, 2.10) | 0.731 |
| Calciumtotal, mg/dL | 8.60 (8.20, 9.00) | 8.60 (8.20, 9.00) | 8.40 (7.90, 9.00) | 0.008 |
| Chloride, mEq/L | 105.0 (102.0, 108.0) | 105.0 (102.0, 108.0) | 105.0 (100.0, 108.0) | 0.718 |
| Glucose, mg/dL | 130 (110, 157) | 127 (109, 150) | 151 (120, 204) | <0.001 |
| Anion gap, mEq/L | 14.0 (12.0, 16.0) | 14.0 (12.0, 16.0) | 16.0 (13.0, 18.0) | <0.001 |
| PT, s | 12.50 (11.68, 13.40) | 12.40 (11.60, 13.20) | 13.00 (12.00, 14.60) | <0.001 |
| APTT, s | 28 (26, 32) | 28 (26, 31) | 29 (26, 33) | 0.017 |
| INR | 1.10 (1.10, 1.20) | 1.10 (1.10, 1.20) | 1.20 (1.10, 1.30) | <0.001 |
| Ureanitrogen, mg/dL | 14 (10, 18) | 13 (10, 17) | 18 (13, 26) | <0.001 |
| Creatinine, mg/dL | 0.80 (0.60, 1.00) | 0.80 (0.60, 0.90) | 0.95 (0.70, 1.40) | <0.001 |
| **Scores** |  |  |  |  |
| SAPSⅡ | 30 (23, 39) | 28 (22, 37) | 40 (34, 52) | <0.001 |
| GCS | 14.0 (12.0, 15.0) | 14.0 (13.0, 15.0) | 15.0 (10.0, 15.0) | 0.006 |
| WFNS grade, n (%) |  |  |  | 0.085 |
| Low (Ⅰ-Ⅲ) | 784 (74.2%) | 648 (75.3%) | 136 (69.4%) |  |
| High (Ⅳ-Ⅴ) | 272 (25.8%) | 212 (24.7%) | 60 (30.6%) |  |
| **Therapy, n (%)** |  |  |  |  |
| Clipping | 35 (3.3%) | 30 (3.5%) | 5 (2.6%) | 0.508 |
| Coiling | 184 (17.4%) | 158 (18.4%) | 26 (13.3%) | 0.089 |
| Ventilation | 797 (75.5%) | 633 (73.6%) | 164 (83.7%) | 0.003 |
| Dobutamine | 9 (0.9%) | 4 (0.5%) | 5 (2.6%) | 0.014 |
| Dopamine | 15 (1.4%) | 8 (0.9%) | 7 (3.6%) | 0.011 |
| Epinephrine | 26 (2.5%) | 12 (1.4%) | 14 (7.1%) | <0.001 |
| Norepinephrine | 200 (18.9%) | 118 (13.7%) | 82 (41.8%) | <0.001 |
| Vasopressin | 90 (8.5%) | 43 (5.0%) | 47 (24.0%) | <0.001 |
| **Outcomes** |  |  |  |  |
| Length of hospital stay, days | 12 (7, 20) | 13 (8, 21) | 5 (2, 13) | <0.001 |
| Length of ICU stay, days | 7 (3, 14) | 8 (3, 14) | 4 (2, 10) | <0.001 |
| ICU mortality, n (%) | 156 (14.8%) | 0 (0.0%) | 156 (79.6%) | <0.001 |
| Median (IQR); n (%); Wilcoxon rank sum test; Pearson's Chi-squared test; Fisher's exact test | | | | |

ICU, Intensive care unit; WBC, white blood cell; RBC, red Blood cell; RDW, red cell distribution width; PT, prothrombin time; APTT, activated partial thromboplastin time; HR, heart rate; SBP, systolic blood pressure; DBP, diastolic blood pressure; MBP, mean arterial pressure; RR, respiratory rate; SpO_2_, percutaneous oxygen saturation; APTT, activated partial thromboplastin time; SAPS Ⅱ, Simplified acute physiology score Ⅱ; GCS, Glasgow coma score; WFNS, World Federation of Neurosurgical Societies; PRR, platelet / red cell distribution width.
