# Supplementary material for: Relationship between baseline platelet-to-red blood cell distribution width ratio and all-cause mortality in non-traumatic subarachnoid hemorrhage: A retrospective analysis of the MIMIC-IV database
Source: PLoS One. 2025 Aug 22;20(8):e0330825. doi: 10.1371/journal.pone.0330825 (PMC12373194; doi:10.1371/journal.pone.0330825)
Supplement: S4 Table — (DOCX) [file pone.0330825.s004.docx]

**S4 Table. Univariate Cox regression analyses for ICU and in-hospital mortality in patients with non-traumatic SAH**

| **Variable** | **ICU mortality** | | | **In-hospital mortality** | | |
| --- | --- | --- | --- | --- | --- | --- |
|  | HR | 95% CI | P-value | HR | 95% CI | P-value |
| Age | 1.55 | 1.10, 2.18 | **0.013** | 1.84 | 1.34, 2.54 | **<0.001** |
| Gender | 1.22 | 0.89, 1.67 | 0.216 | 1.25 | 0.94, 1.66 | 0.126 |
| Race |  |  |  |  |  |  |
| White | - | - | -- | - | - | - |
| Black | 1.35 | 0.64, 2.84 | 0.426 | 1.10 | 0.59, 2.08 | 0.759 |
| Asian | 2.03 | 1.00, 4.13 | 0.050 | 1.41 | 0.71, 2.83 | 0.328 |
| Other | 2.43 | 1.72, 3.41 | **<0.001** | 2.06 | 1.53, 2.78 | **<0.001** |
| Hypertension | 0.62 | 0.45, 0.86 | 0.004 | 0.76 | 0.58, 1.01 | 0.060 |
| Diabetes | 0.99 | 0.67, 1.45 | 0.941 | 1.17 | 0.84, 1.62 | 0.356 |
| Heart failure | 1.94 | 1.21, 3.10 | **0.006** | 1.57 | 1.02, 2.41 | **0.039** |
| Myocardial infarction | 2.29 | 1.31, 3.99 | **0.003** | 2.29 | 1.39, 3.78 | **0.001** |
| Malignant tumor | 0.70 | 0.36, 1.37 | 0.293 | 0.79 | 0.46, 1.36 | 0.391 |
| Chronic kidney disease | 2.42 | 1.48, 3.96 | **<0.001** | 1.97 | 1.26, 3.07 | **0.003** |
| Cirrhosis | 3.31 | 1.79, 6.12 | **<0.001** | 2.66 | 1.55, 4.55 | **<0.001** |
| Pneumonia | 0.87 | 0.61, 1.26 | 0.468 | 1.04 | 0.76, 1.43 | 0.797 |
| Hyperlipoidemia | 1.09 | 0.77, 1.54 | 0.633 | 1.20 | 0.88, 1.63 | 0.241 |
| WBC | 1.05 | 1.03, 1.07 | **<0.001** | 1.05 | 1.03, 1.07 | **<0.001** |
| RBC | 0.83 | 0.65, 1.07 | 0.150 | 0.91 | 0.73, 1.12 | 0.356 |
| Platelet | 1.00 | 0.99, 1.00 | **<0.001** | 1.00 | 0.99, 1.00 | **<0.001** |
| Hemoglobin | 0.93 | 0.86, 1.02 | 0.114 | 0.96 | 0.90, 1.03 | 0.282 |
| RDW | 1.24 | 1.16, 1.33 | **<0.001** | 1.20 | 1.13, 1.27 | **<0.001** |
| Sodium | 1.05 | 1.01, 1.09 | **0.012** | 1.04 | 1.01, 1.08 | **0.024** |
| Potassium | 1.48 | 1.23, 1.78 | **<0.001** | 1.40 | 1.19, 1.64 | **<0.001** |
| Magnesium | 1.05 | 0.78, 1.41 | 0.740 | 0.90 | 0.59, 1.36 | 0.601 |
| Calciumtotal | 0.80 | 0.64, 0.99 | 0.045 | 0.84 | 0.69, 1.03 | 0.091 |
| Chloride | 1.00 | 0.97, 1.03 | >0.999 | 0.99 | 0.96, 1.02 | 0.619 |
| Glucose | 1.00 | 1.00, 1.01 | **<0.001** | 1.00 | 1.00, 1.01 | **<0.001** |
| Aniongap | 1.15 | 1.12, 1.19 | **<0.001** | 1.14 | 1.11, 1.17 | **<0.001** |
| PT | 1.06 | 1.04, 1.08 | **<0.001** | 1.04 | 1.02, 1.06 | **<0.001** |
| APTT | 1.01 | 1.00, 1.01 | 0.112 | 1.00 | 1.00, 1.01 | 0.235 |
| INR | 1.96 | 1.64, 2.36 | **<0.001** | 1.55 | 1.31, 1.82 | **<0.001** |
| Ureanitrogen | 1.03 | 1.02, 1.04 | **<0.001** | 1.03 | 1.02, 1.03 | **<0.001** |
| Creatinine | 1.18 | 1.10, 1.25 | **<0.001** | 1.19 | 1.12, 1.26 | **<0.001** |
| Clipping | 0.60 | 0.22, 1.63 | 0.321 | 0.67 | 0.28, 1.63 | 0.376 |
| Coiling | 0.61 | 0.39, 0.96 | **0.031** | 0.64 | 0.43, 0.97 | **0.037** |
| Ventilation | 0.75 | 0.49, 1.15 | 0.189 | 1.01 | 0.69, 1.50 | 0.942 |
| Heart Rate | 1.02 | 1.01, 1.02 | **<0.001** | 1.01 | 1.00, 1.02 | **0.007** |
| SBP | 0.99 | 0.99, 1.00 | 0.062 | 1.00 | 0.99, 1.00 | 0.155 |
| DBP | 1.00 | 0.99, 1.01 | 0.874 | 1.00 | 0.99, 1.00 | 0.380 |
| MAP | 1.00 | 0.99, 1.01 | 0.405 | 1.00 | 0.99, 1.01 | 0.428 |
| RR | 1.04 | 1.02, 1.07 | **0.001** | 1.04 | 1.02, 1.06 | **0.001** |
| SpO_2_ | 1.00 | 0.97, 1.04 | 0.822 | 1.01 | 0.97, 1.05 | 0.539 |
| Temperature | 0.89 | 0.85, 0.92 | **<0.001** | 0.88 | 0.85, 0.91 | **<0.001** |
| Dobutamine | 2.46 | 1.01, 6.02 | **0.048** | 2.47 | 1.02, 6.01 | **0.046** |
| Dopamine | 2.57 | 1.13, 5.83 | **0.024** | 2.81 | 1.32, 5.99 | **0.007** |
| Epinephrine | 2.47 | 1.41, 4.32 | **0.002** | 2.06 | 1.18, 3.60 | **0.011** |
| Norepinephrine | 2.38 | 1.72, 3.29 | **<0.001** | 2.39 | 1.79, 3.19 | **<0.001** |
| Vasopressin | 3.10 | 2.17, 4.42 | **<0.001** | 3.07 | 2.20, 4.28 | **<0.001** |
| Sepsis | 1.37 | 0.95, 1.98 | 0.090 | 1.53 | 1.11, 2.11 | 0.009 |
| SAPS Ⅱ | 3.81 | 2.73, 5.30 | **<0.001** | 3.31 | 2.44, 4.50 | **<0.001** |
| GCS | 0.70 | 0.48, 1.03 | 0.071 | 0.69 | 0.49, 0.98 | 0.036 |
| WFNS grade | 1.09 | 0.77, 1.53 | 0.631 | 1.11 | 0.82, 1.50 | 0.508 |
| Charlson comorbidity index | 1.14 | 1.08, 1.20 | **<0.001** | 1.14 | 1.09, 1.20 | **<0.001** |

ICU, Intensive care unit; WBC, white blood cell; RBC, red Blood cell; RDW, red cell distribution width; PT, prothrombin time; APTT, activated partial thromboplastin time; HR, heart rate; SBP, systolic blood pressure; DBP, diastolic blood pressure; MBP, mean arterial pressure; RR, respiratory rate; SpO_2_, percutaneous oxygen saturation; APTT, activated partial thromboplastin time; SAPS Ⅱ, Simplified acute physiology score Ⅱ; GCS, Glasgow coma score; WFNS, World Federation of Neurosurgical Societies; PRR, platelet / red cell distribution width; HR, hazard ratio; CI, confidence interval.
