# Supplementary material for: Relationship between baseline platelet-to-red blood cell distribution width ratio and all-cause mortality in non-traumatic subarachnoid hemorrhage: A retrospective analysis of the MIMIC-IV database
Source: PLoS One. 2025 Aug 22;20(8):e0330825. doi: 10.1371/journal.pone.0330825 (PMC12373194; doi:10.1371/journal.pone.0330825)
Supplement: S5 Table — (DOCX) [file pone.0330825.s005.docx]

**S5 Table. Sensitivity analysis: subgroup analysis of PRR threshold [ICU mortality]**

| Subgroup | **< 22.6** | **≥ 22.6** | HR (95% CI) | P value | P for interaction |
| --- | --- | --- | --- | --- | --- |
| Age |  |  |  |  | **0.5** |
| ˂ 60 | 38/351 (10.8) | 8/62 (12.9) | 1.00 (0.46-2.16) | 0.995 |  |
| ≥ 60 | 101/576 (17.5) | 9/67 (13.4) | 0.69 (0.35-1.37) | 0.29 |  |
| Gender |  |  |  |  | **0.021** |
| Female | 73/511 (14.3) | 7/88 (8.0) | 0.46 (0.21-1.00) | 0.051 |  |
| Male | 66/416 (15.9) | 10/41 (24.4) | 1.47 (0.75-2.86) | 0.257 |  |
| Race |  |  |  |  | **0.056** |
| White | 50/529 (9.5) | 5/80 (6.2) | 0.57 (0.23-1.44) | 0.236 |  |
| Asian | 9/38 (23.7) | 0/5 (0.0) | 0.00 (0.00-Inf) | 0.999 |  |
| Black | 8/64 (12.5) | 0/10 (0.0) | 0.00 (0.00-Inf) | 0.998 |  |
| Other | 72/296 (24.3) | 12/34 (35.3) | 1.30 (0.70-2.40) | 0.41 |  |
| Hypertension |  |  |  |  | **0.333** |
| No | 82/460 (17.8) | 8/61 (13.1) | 0.60 (0.29-1.24) | 0.167 |  |
| Yes | 57/467 (12.2) | 9/68 (13.2) | 1.01 (0.50-2.05) | 0.972 |  |
| Diabetes |  |  |  |  | **0.775** |
| No | 109/729 (15.0) | 15/111 (13.5) | 0.80 (0.47-1.38) | 0.427 |  |
| Yes | 30/198 (15.2) | 2/18 (11.1) | 0.66 (0.16-2.78) | 0.574 |  |
| Heart failure |  |  |  |  | **0.781** |
| No | 120/855 (14.0) | 16/126 (12.7) | 0.80 (0.47-1.35) | 0.403 |  |
| Yes | 19/72 (26.4) | 1/3 (33.3) | 1.06 (0.14-7.99) | 0.957 |  |
| Coiling |  |  |  |  | **0.934** |
| No | 120/777 (15.4) | 13/95 (13.7) | 0.81 (0.45-1.43) | 0.463 |  |
| Yes | 19/150 (12.7) | 4/34 (11.8) | 0.84 (0.28-2.50) | 0.752 |  |
| Sepsis |  |  |  |  | **0.558** |
| No | 41/486 (8.4) | 3/60 (5.0) | 0.60 (0.18-1.93) | 0.389 |  |
| Yes | 98/441 (22.2) | 14/69 (20.3) | 0.82 (0.47-1.44) | 0.494 |  |
| SAPS Ⅱ |  |  |  |  | **0.98** |
| ˂ 45 | 91/821 (11.1) | 11/113 (9.7) | 0.75 (0.40-1.41) | 0.375 |  |
| ≥ 45 | 48/106 (45.3) | 6/16 (37.5) | 0.75 (0.32-1.76) | 0.511 |  |
| GCS |  |  |  |  | **0.343** |
| ˂ 9 | 31/124 (25.0) | 3/19 (15.8) | 0.52 (0.16-1.71) | 0.284 |  |
| ≥ 9 | 108/803 (13.4) | 14/110 (12.7) | 0.86 (0.49-1.50) | 0.599 |  |
| no. of events / total no. (%) | | | | | |
