# Supplementary material for: Relationship between baseline platelet-to-red blood cell distribution width ratio and all-cause mortality in non-traumatic subarachnoid hemorrhage: A retrospective analysis of the MIMIC-IV database
Source: PLoS One. 2025 Aug 22;20(8):e0330825. doi: 10.1371/journal.pone.0330825 (PMC12373194; doi:10.1371/journal.pone.0330825)
Supplement: S6 Table — (DOCX) [file pone.0330825.s006.docx]

**S6 Table. Sensitivity analysis: subgroup analysis of PRR threshold [Hospital mortality]**

| Subgroup | < 22.6 | ≥ 22.6 | HR (95% CI) | P value | P for interaction |
| --- | --- | --- | --- | --- | --- |
| Age |  |  |  |  | **0.206** |
| ˂ 60 | 41/351 (11.7) | 9/62 (14.5) | 1.20 (0.58-2.46) | 0.628 |  |
| ≥ 60 | 135/576 (23.4) | 11/67 (16.4) | 0.66 (0.36-1.23) | 0.191 |  |
| Gender |  |  |  |  | **0.033** |
| Female | 86/511 (16.8) | 9/88 (10.2) | 0.53 (0.27-1.05) | 0.068 |  |
| Male | 90/416 (21.6) | 11/41 (26.8) | 1.43 (0.76-2.68) | 0.269 |  |
| Race |  |  |  |  | **0.021** |
| White | 73/529 (13.8) | 7/80 (8.8) | 0.56 (0.26-1.23) | 0.15 |  |
| Asian | 9/38 (23.7) | 0/5 (0.0) | 0.00 (0.00-Inf) | 0.998 |  |
| Black | 11/64 (17.2) | 0/10 (0.0) | 0.00 (0.00-Inf) | 0.998 |  |
| Other | 83/296 (28.0) | 13/34 (38.2) | 1.50 (0.83-2.69) | 0.177 |  |
| Hypertension |  |  |  |  | **0.467** |
| No | 97/460 (21.1) | 9/61 (14.8) | 0.67 (0.34-1.32) | 0.246 |  |
| Yes | 79/467 (16.9) | 11/68 (16.2) | 0.93 (0.49-1.75) | 0.818 |  |
| Diabetes |  |  |  |  | **0.875** |
| No | 132/729 (18.1) | 17/111 (15.3) | 0.81 (0.49-1.35) | 0.418 |  |
| Yes | 44/198 (22.2) | 3/18 (16.7) | 0.75 (0.23-2.42) | 0.63 |  |
| Heart failure |  |  |  |  | **0.764** |
| No | 153/855 (17.9) | 19/126 (15.1) | 0.80 (0.50-1.30) | 0.371 |  |
| Yes | 23/72 (31.9) | 1/3 (33.3) | 1.13 (0.15-8.45) | 0.903 |  |
| Coiling |  |  |  |  | **0.557** |
| No | 155/777 (19.9) | 15/95 (15.8) | 0.77 (0.45-1.30) | 0.325 |  |
| Yes | 21/150 (14.0) | 5/34 (14.7) | 1.07 (0.40-2.85) | 0.886 |  |
| Sepsis |  |  |  |  | **0.191** |
| No | 55/486 (11.3) | 3/60 (5.0) | 0.43 (0.13-1.38) | 0.156 |  |
| Yes | 121/441 (27.4) | 17/69 (24.6) | 0.93 (0.56-1.55) | 0.779 |  |
| SAPS Ⅱ |  |  |  |  | **0.578** |
| ˂ 45 | 123/821 (15.0) | 13/113 (11.5) | 0.72 (0.41-1.28) | 0.268 |  |
| ≥ 45 | 53/106 (50.0) | 7/16 (43.8) | 0.92 (0.42-2.04) | 0.846 |  |
| GCS |  |  |  |  | **0.63** |
| ˂ 9 | 36/124 (29.0) | 4/19 (21.1) | 0.63 (0.22-1.78) | 0.382 |  |
| ≥ 9 | 140/803 (17.4) | 16/110 (14.5) | 0.82 (0.49-1.38) | 0.457 |  |
| no. of events / total no. (%) | | | | | |
