# Supplementary material for: Relationship between baseline platelet-to-red blood cell distribution width ratio and all-cause mortality in non-traumatic subarachnoid hemorrhage: A retrospective analysis of the MIMIC-IV database
Source: PLoS One. 2025 Aug 22;20(8):e0330825. doi: 10.1371/journal.pone.0330825 (PMC12373194; doi:10.1371/journal.pone.0330825)
Supplement: Attachment — Submitted filename: Response to Reviewers.docx [file pone.0330825.s008.docx]

Dear Editor and Reviewers,

Many thanks to the editor and reviewers for their advice and recognition of the manuscript.We appreciate your insightful comments and suggestions. Those comments are all valuable and very helpful for revising and improving our paper, as well as the important guiding significance to our researches. We have carefully studied the comments and made corrections, which we hope will meet your approval. The corresponding modification positions in the revised manuscript are listed in the responses below. In addition, the revised paragraphs are marked with different colors (**Blue** for Reviewer 1 and **Red** for Reviewer 2). We shall now address the comments and suggestions point by point.

**For Editor:**

1. Please ensure that your manuscript meets PLOS ONE's style requirements, including those for file naming.

**Response:** Thanks for the reminder. Yes, we have ensured the manuscript fully meets PLOS ONE's style requirements, including file naming, and have made any necessary adjustments.

2. Thank you for stating the following financial disclosure: Health Commission of Henan Province (grant numbers: SBGJ202402008 ). Please state what role the funders took in the study. If the funders had no role, please state: "The funders had no role in study design, data collection and analysis, decision to publish, or preparation of the manuscript." If this statement is not correct you must amend it as needed. Please include this amended Role of Funder statement in your cover letter; we will change the online submission form on your behalf.

**Response:** Yes, we have included the Role of Funder statement in our cover letter.

**Funding statement:**

This work was supported by the Health Commission of Henan Province (grant number: SBGJ202402008). The funders had no role in study design, data collection and analysis, decision to publish, or preparation of the manuscript. Guang Feng (the funding recipient) contributed to study design, data collection and analysis, and manuscript preparation.

3.Your abstract cannot contain citations. Please only include citations in the body text of the manuscript, and ensure that they remain in ascending numerical order on first mention.

**Response:** Yes, we have made sure that there are no citations in the abstract.

4. Please remove all personal information, ensure that the data shared are in accordance with participant consent, and re-upload a fully anonymized data set. Note: spreadsheet columns with personal information must be removed and not hidden as all hidden columns will appear in the published file.

**Response:** Yes, we have made sure that the manuscript does not contain any personal information.

5. Please include captions for your Supporting Information files at the end of your manuscript, and update any in-text citations to match accordingly.

**Response:** Yes, we have added captions to the Supporting Information files at the end of the manuscript and updated the in-text citations to match.

**For Reviewer #1:**

**1.** In the Introduction, the author described, "Early brain injury, which occurs within the first 72 h after SAH, is characterized by a cascade of inflammatory responses, oxidative stress, and neuronal apoptosis. Targeting these pathological mechanisms may improve outcomes and reduce mortality in patients with non-traumatic SAH." The authors should further elaborate on the potential association between PRR and these pathophysiological mechanisms, which would provide more referral for the presentation of PRR.

**Response:** Thank you for your valuable comment. We acknowledge the importance of further exploring the potential association between the PRR and the pathophysiological mechanisms of early brain injury (EBI) after SAH as you suggested. We elaborated the specific details in the discussion, so we briefly discussed the relationship between PRR and EBI in the introduction.

Page 4 Line 88-96: “After SAH, platelets release substances that drive inflammation, and a higher PRR may indicate a more active platelet-mediated inflammatory response. A low PRR can result from pathological thrombocytopenia. Platelets generate reactive oxygen species (ROS), causing oxidative damage, while red blood cells have antioxidant functions; changes in PRR reflect oxidative stress imbalances. Inflammatory cytokines and oxidative stress after SAH trigger neuronal apoptosis, and platelets are involved through interactions with the blood brain barrier. PRR may serve as a biomarker for the balance between platelet-related pro-apoptotic effects and the red blood cell state.”

**2.** In the Methods, the authors included all baseline information in the regression analysis as a potential confounding factor, which may result in the greatest reduction in confounding bias. However, the presence of multicollinearity can distort the real effect. Therefore, the authors need to perform multicollinearity analyses to exclude variables that should not be included in the regression analysis.

**Response:** According to your insightful suggestion, we immediately carried out collinearity analysis, and used the methods of variance expansion factor (VIF) and tolerance to detect the degree of collinearity between variables. For the variables with VIF > 5 and tolerance < 0.2, we decided to exclude them from the regression analysis to ensure the accuracy and reliability of the regression results.

Page 7 Line 170-172: “(3) Variables with variance inflation factor (VIF) > 10 and tolerance < 0.1 were considered to have serious collinearity and were excluded. The details of the VIFs were shown in **S2 Table**.”

We describe the process of collinearity analysis in detail in the methodology section. The variables finally included in COX regression model II include:

Page 7 Line 175-178: “age, gender, race, heart failure, malignant tumor, chronic kidney disease, cirrhosis, pneumonia, WBC, urea nitrogen, creatinine, coiling, dobutamine, norepinephrine, vasopressin, sepsis, and charlson comorbidity index.”

**S2 Table. Variance Inflation Factor and Tolerance**

| Term | ICU mortality | | In-hospital mortality | |
| --- | --- | --- | --- | --- |
|  | VIF | Tolerance | VIF | Tolerance |
| Gender | 1.536527 | 0.650818312 | 1.427104 | 0.700719689 |
| Race | 2.365386 | 0.422763895 | 2.029596 | 0.492708795 |
| Hypertension | 1.432434 | 0.698112418 | 1.439963 | 0.694462141 |
| Age | 2.283043 | 0.4380120 | 2.119726 | 0.4717592 |
| Diabetes | 1.695731 | 0.589716143 | 1.706443 | 0.586014380 |
| Heart failure | 1.839372 | 0.543663879 | 1.753142 | 0.570404365 |
| Myocardial infarction | 1.963212 | 0.509369336 | 1.835009 | 0.544956312 |
| Malignant tumor | 1.366298 | 0.731904572 | 1.222219 | 0.818183984 |
| Chronic kidney disease | 2.163563 | 0.462200506 | 2.000120 | 0.499970005 |
| Cirrhosis | 2.503507 | 0.399439718 | 2.168552 | 0.461137109 |
| Pneumonia | 1.440453 | 0.694226200 | 1.453815 | 0.687845371 |
| Hyperlipoidemia | 1.348539 | 0.741543447 | 1.246881 | 0.802001170 |
| Sepsis | 1.961714 | 0.509758304 | 1.854806 | 0.539139896 |
| Charlson | 2.675928 | 0.373702105 | 2.586723 | 0.386589466 |
| WBC | 1.679360 | 0.595464950 | 1.655348 | 0.604102727 |
| RBC | 10.788064 | 0.092695036 | 9.513165 | 0.105117482 |
| Platelet count | 2.237795 | 0.446868440 | 2.102796 | 0.475557248 |
| Hemoglobin | 11.269468 | 0.088735334 | 10.458109 | 0.095619577 |
| RDW | 2.709901 | 0.369017111 | 2.392885 | 0.417905615 |
| Sodium | 5.485718 | 0.182291527 | 4.511714 | 0.221645263 |
| Potassium | 2.628138 | 0.380497560 | 2.262782 | 0.441933819 |
| Magnesium | 1.691942 | 0.591036810 | 2.014803 | 0.496326552 |
| Calciumtotal | 2.321924 | 0.430677397 | 5.424522 | 0.184348046 |
| Chloride | 6.312372 | 0.158419061 | 1.841474 | 0.543043195 |
| Glucose | 1.813140 | 0.551529384 | 2.912777 | 0.343315033 |
| Aniongap | 3.476604 | 0.287637009 | 330.309633 | 0.003027462 |
| PT | 335.231286 | 0.002983015 | 1.442556 | 0.693214088 |
| APTT | 1.600283 | 0.624889636 | 334.623342 | 0.002988435 |
| INR | 339.091237 | 0.002949059 | 3.260740 | 0.306678829 |
| Ureanitrogen | 3.000060 | 0.333326645 | 2.453768 | 0.407536478 |
| Creatinine | 2.584507 | 0.386920925 | 1.144887 | 0.873448811 |
| Clipping | 1.138728 | 0.878173024 | 1.391610 | 0.718592361 |
| Coiling | 1.481927 | 0.674797067 | 1.447143 | 0.691016616 |
| Ventilation | 1.479912 | 0.675715672 | 1.677032 | 0.596291443 |
| HR | 1.710602 | 0.584589352 | 5.110447 | 0.195677604 |
| SBP | 8.444855 | 0.118415293 | 15.444101 | 0.064749642 |
| DBP | 39.184739 | 0.025520139 | 24.704031 | 0.040479223 |
| MBP | 62.020671 | 0.016123657 | 1.482235 | 0.674656653 |
| RR | 1.483955 | 0.673874665 | 1.366563 | 0.731762848 |
| SpO2 | 1.353299 | 0.738934779 | 1.667848 | 0.599574944 |
| Temperature | 1.529641 | 0.653748140 | 1.587428 | 0.629949789 |
| Dobutamine | 1.855976 | 0.538800033 | 1.752084 | 0.570748842 |
| Dopamine | 1.799186 | 0.555806983 | 1.834909 | 0.544986286 |
| Epinephrine | 2.137662 | 0.467800836 | 2.193704 | 0.455850113 |
| Norepinephrine | 2.132437 | 0.468947127 | 2.433540 | 0.410923930 |
| Vasopressin | 2.596180 | 0.385181334 | 5.145403 | 0.194348235 |
| SAPS Ⅱ | 5.950259 | 0.168059901 | 9.579483 | 0.104389768 |
| GCS | 11.004872 | 0.090868846 | 6.301759 | 0.158685849 |
| WFNS grade | 7.355099 | 0.135960102 | 1.532007 | 0.652738510 |

**Table 2. Multivariable cox regression models evaluating the association between PRR and ICU and hospital all-cause mortality.**

| **Variable** | **Crude** | | **Model Ⅰ** | | **Model Ⅱ** | |
| --- | --- | --- | --- | --- | --- | --- |
|  | HR (95% CI) | p-value | HR (95% CI) | p-value | HR (95% CI) | p-value |
| Hospital all-cause mortality |  | | | | | |
| Q1(≤12.67) | 1 (Reference) |  | 1 (Reference) |  | 1 (Reference) |  |
| Q2(12.68-15.99) | 0.46 (0.31, 0.67) | <0.001 | 0.49 (0.34, 0.72) | <0.001 | 0.61 (0.40, 0.92) | 0.017 |
| Q3(16.00-19.41) | 0.44 (0.29, 0.65) | <0.001 | 0.49 (0.33, 0.74) | <0.001 | 0.60 (0.39, 0.92) | 0.020 |
| Q4(≥19.42) | 0.43 (0.29, 0.64) | <0.001 | 0.58 (0.38, 0.86) | 0.007 | 0.60 (0.39, 0.92) | 0.019 |
| P for trend |  | <0.001 |  | 0.001 |  | 0.015 |
| ICU all-cause mortality |  |  |  |  |  |  |
| Q1(≤12.67) | 1 (Reference) |  | 1 (Reference) |  | 1 (Reference) |  |
| Q2(12.68-15.99) | 0.40 (0.26, 0.62) | <0.001 | 0.42 (0.27, 0.64) | <0.001 | 0.54 (0.34, 0.86) | 0.009 |
| Q3(16.00-19.41) | 0.40 (0.26, 0.63) | <0.001 | 0.43 (0.28, 0.68) | <0.001 | 0.55 (0.34, 0.90) | 0.016 |
| Q4(≥19.42) | 0.40 (0.26, 0.61) | <0.001 | 0.51 (0.33, 0.79) | 0.002 | 0.54 (0.34, 0.87) | 0.010 |
| P for trend |  | <0.001 |  | <0.001 |  | 0.010 |
| Crude model: adjusted for none.  Model I: adjusted for Age, Gender, and Race.  Model Ⅱ: adjusted for Age, Gender, Race, Heart failure, Malignant tumor, Chronic kidney disease, Cirrhosis, Pneumonia, WBC, Ureanitrogen, Creatinine, Coiling, Dobutamine, Norepinephrine, Sepsis, and Charlson comorbidity index. | | | | | | |

WBC, white blood cell; PRR, platelet / red cell distribution width; HR, hazard ratio; CI, confidence interval.

**3.** In figure4, the abscissa range of subgroup analyses should be narrowed, not from zero to two. This will make it more aesthetically pleasing.

**Response:** Thank you for your feedback on Figure 4. Upon careful consideration, we adjust the abscissa range in Figure 4. By narrowing the range, we can better present the data in the subgroup analyses, making the figure more aesthetically pleasing and facilitating a more straightforward interpretation of the results.

**Fig 4.** The relationship between PRR and (A) ICU mortality and (B) in-hospital mortality in subgroup analysis.

**4.** The authors discussed in detail the possible mechanisms of the association between PRR and outcomes, and cited some of the advantages of this study. However, on this basis, the authors should discuss the novel findings of this study.

**Response:** We appreciate your insightful suggestion. The novel findings of this study indeed deserve a more in-depth discussion.

Page 12 Line 319-332: “Our study presents several novel and significant findings regarding the PRR in non-traumatic SAH. Firstly, we identified PRR as a potential biomarker for non-traumatic SAH, pioneering the exploration of this combined ratio in relation to patient outcomes, unlike prior studies that focused on its individual components separately. This offers fresh insights into SAH pathophysiology, highlighting the complex and crucial interplay between platelet- and red blood cell-related factors.We discovered a significant inverse association between admission PRR and the risk of ICU and in-hospital mortality in non-traumatic SAH patients. Patients with low PRR levels had higher mortality and shorter survival time. In addition, there was a nonlinear correlation between PRR and all-cause mortality at admission. Notably, the threshold effect observed at PRR=22.6 suggests a dichotomous prognostic role: below this critical value, each unit increase in PRR correlates with reduced all-cause mortality risk, whereas no such association exists above the threshold. This nonlinear pattern may reflect dual pathophysiological mechanisms. We also found no interaction between different subgroups in the correlation between PRR level and ICU and all-cause mortality.”

**For Reviewer #2:**

**1.** Expand Discussion on Limitations: Acknowledge the retrospective design’s inability to establish causality and the potential impact of unmeasured confounders (e.g., aneurysm characteristics, timing of intervention). Discuss the lack of dynamic PRR data and its implications for clinical practice.

**Response:** In the discussion part of the revised manuscript, we emphasize the limitations of retrospective research design in causal inference, and list in detail the potential influence of unmeasured confounding factors on the research results. At the same time, we explain that the inability to obtain dynamic information may lead to some limitations in clinical practice, and point out that future research needs to pay attention to this issue.

Page 15 Line 416-421: “Because the dynamic change information of PRR can not be obtained, there may be some limitations in clinical practice, such as whether to carry out platelet transfusion and when to carry out treatment. Future prospective trials should evaluate whether PRR-guided interventions (e.g., early platelet transfusion for PRR<22.6) improve outcomes.”

Page 15 Line 421-424: “For example, the specific location of aneurysm characteristics (location, size and shape) and the potential impact of intervention timing (treatment at different times after bleeding) on the research results.”

**2.** Enhance Transparency in Methods: Clarify how missing platelet/RDW data were handled (e.g., exclusion criteria, imputation methods). Justify the exclusion of patients with ICU stays <24 hours and assess sensitivity to this criterion.

**Response:** We thank the reviewer for the careful reminder. We included a total of 1077 patients, of whom only 21 patients were excluded due to lack of platelet and RDW data. The elimination process is fully shown in our flow chart (**Figure 1**). Other missing variables > 5% are excluded to ensure the accuracy of data analysis. Missing data are interpolated by k-nearest neighbors (KNN). Details of missing data are shown in **S1 Table**. Patients who stay in ICU for less than 24 hours may be discharged prematurely due to mild illness, or the treatment and follow-up process may be incomplete due to the death of patients, which will affect the accuracy of the research results.

Page 5 Line 130: “Details of missing data are shown in S1 Table.”

Page 7 Line 186-187: “Missing data are interpolated by k-nearest neighbors (KNN).”

| **S1 Table.** Details of missing values. | | |
| --- | --- | --- |
| **Variables** | **The number of missing values** | **The percent of missing values** |
| Calciumtotal | 1 | <0.1% |
| Magnesium | 2 | 0.1% |
| PT | 25 | 2.3% |
| APTT | 28 | 2.6% |
| SBP | 20 | 1.8% |
| DBP | 20 | 1.8% |
| MAP | 20 | 1.8% |
| GCS | 1 | <0.1% |

Note: PT, Prothrombin Time; APTT, Activated Partial Thromboplastin Time; SBP, Systolic Blood Pressure; DBP, Diastolic Blood Pressure; MAP, mean arterial pressure; GCS, Glasgow Coma Scale.

**3.**Strengthen Subgroup Analyses: Report power calculations for subgroup interactions to contextualize non-significant findings. Highlight racial / ethnic disparities in baseline characteristics (e.g., Table 1) and discuss implications.

**Response:** Thank you for asking this question. In subgroup analysis, we calculated the statistical efficacy of PRR interacting with different subgroups (age, sex, race, hypertension, diabetes, heart failure, interventional embolization, sepsis, SAPS Ⅱ, GCS). Table 4 and Figure 4 show the detailed results of power calculations of subgroup analysis. In order to explore whether there is any difference in mortality between races in the level of admission PRR, the interaction calculation results of race subgroups are added to the subgroup analysis. In the discussion section, we evaluate the potential impact of racial differences on the research results.

Page 12 Line 332-337: “In the subgroup analysis, there was no significant interaction between admission PRR level and race in ICU mortality, but a significant one in hospitalization mortality. We suspect that the small number of Asian participants affects the results. Also, comorbidities in certain racial groups that affect platelet or red blood cell function may confound the association between PRR and SAH outcomes, yet current evidence can't prove this.”

**Table 4. The subgroup analysis for baseline PRR on ICU and hospital mortality.**

| Subgroup | N | ICU mortality | | In-hospital mortality | |
| --- | --- | --- | --- | --- | --- |
|  |  | HR (95% CI) | P for interaction | HR (95% CI) | P for interaction |
| Overall | 1056 | 0.93 (0.90-0.95) |  | 0.94 (0.92-0.96) |  |
| Age |  |  | **0.502** |  | **0.818** |
| ˂ 60 | 413 | 0.92 (0.87-0.97) |  | 0.94 (0.89-0.98) |  |
| ≥ 60 | 643 | 0.94 (0.91-0.97) |  | 0.94 (0.92-0.97) |  |
| Gender |  |  | **0.434** |  | **0.315** |
| Female | 599 | 0.92 (0.88-0.95) |  | 0.93 (0.90-0.96) |  |
| Male | 457 | 0.94 (0.90-0.98) |  | 0.95 (0.92-0.99) |  |
| Race |  |  | **0.105** |  | **0.005** |
| White | 609 | 0.90 (0.86-0.94) |  | 0.92 (0.89-0.95) |  |
| Asian | 43 | 0.96 (0.85-1.07) |  | 0.98 (0.88-1.09) |  |
| Black | 74 | 0.85 (0.74-0.99) |  | 0.82 (0.71-0.93) |  |
| Other | 330 | 0.96 (0.92-1.00) |  | 0.98 (0.94-1.01) |  |
| Hypertension |  |  | **0.127** |  | **0.212** |
| No | 521 | 0.91 (0.88-0.94) |  | 0.93 (0.90-0.96) |  |
| Yes | 535 | 0.95 (0.91-0.99) |  | 0.96 (0.92-0.99) |  |
| Diabetes |  |  | **0.167** |  | **0.322** |
| No | 840 | 0.92 (0.89-0.95) |  | 0.93 (0.91-0.96) |  |
| Yes | 216 | 0.96 (0.91-1.02) |  | 0.97 (0.92-1.01) |  |
| Heart failure |  |  | **0.233** |  | **0.158** |
| No | 981 | 0.93 (0.90-0.95) |  | 0.94 (0.91-0.96) |  |
| Yes | 75 | 0.97 (0.90-1.05) |  | 0.99 (0.92-1.06) |  |
| Coiling |  |  | **0.859** |  | **0.858** |
| No | 872 | 0.93 (0.90-0.96) |  | 0.94 (0.92-0.97) |  |
| Yes | 184 | 0.94 (0.88-1.01) |  | 0.94 (0.88-1.01) |  |
| Sepsis |  |  | **0.033** |  | **0.017** |
| No | 546 | 0.88 (0.83-0.93) |  | 0.90 (0.85-0.95) |  |
| Yes | 510 | 0.94 (0.91-0.97) |  | 0.96 (0.93-0.98) |  |
| SAPS Ⅱ |  |  | **0.748** |  | **0.317** |
| ˂ 45 | 934 | 0.95 (0.91-0.98) |  | 0.95 (0.92-0.97) |  |
| ≥ 45 | 122 | 0.94 (0.91-0.98) |  | 0.97 (0.93-1.00) |  |
| GCS |  |  | **0.623** |  | **0.901** |
| ˂ 9 | 143 | 0.92 (0.87-0.98) |  | 0.94 (0.89-0.99) |  |
| ≥ 9 | 913 | 0.93 (0.90-0.96) |  | 0.94 (0.92-0.97) |  |

PRR, platelet / red cell distribution width; ICU, Intensive care unit; SAPS Ⅱ, Simplified Acute Physiology Score; GCS, Glasgow Coma Scale; HR, hazard ratio; CI, confidence interval.

**Fig 4.** The relationship between PRR and (A) ICU mortality and (B) in-hospital mortality in subgroup analysis.

**4.**Reinterpret Threshold Findings: Conduct sensitivity analyses to test the robustness of the PRR threshold (22.6) across subgroups. Provide mechanistic hypotheses for the observed threshold (e.g., platelet function vs. RBC fragility).

**Response:** Thank you for your constructive doubts. We carried out sensitivity analysis to test the robustness of PRR threshold (22.6) in different subgroups. Sensitivity analysis showed that the interaction between different subgroups was not significant (p value of interaction > 0.05), except for gender and race. This is because the population distribution has changed after threshold grouping, and prospective cohort study should be conducted in the future to further confirm its consistency. However, on the whole, the threshold of admission PRR (22.6) is robust in different subgroups. For the observed threshold, we propose a mechanism hypothesis based on existing research. From the aspect of platelet function, the change of platelet quantity or activity around the threshold may have an influence on coagulation function and inflammatory reaction; From the point of view of RBC fragility, the stability of erythrocytes at different PRR levels is inconsistent and affects the oxygen transport function.

Page 11-12 Line 315-317: “Sensitivity analysis showed that the interaction was not significant in different subgroups, except for gender and race, which proved the robustness of PRR threshold (p for interaction > 0.05)(S5 and S6 Tables).”

Page 12 Line 337-346: “Sensitivity analysis showed that the interaction between different subgroups was not significant (p value of interaction > 0.05), except for gender and race. This is because the population distribution has changed after threshold grouping, and prospective cohort study should be conducted in the future to further confirm its consistency. However, on the whole, the threshold of admission PRR (22.6) is robust in different subgroups. From the aspect of platelet function, the change of platelet quantity or activity around the threshold may have an influence on coagulation function and inflammatory reaction; From the point of view of RBC fragility, the stability of erythrocytes at different PRR levels is inconsistent and affects the oxygen transport function.”

S5 Table. Sensitivity analysis: subgroup analysis of PRR threshold [ICU mortality]

| Subgroup | **< 22.6** | **≥ 22.6** | HR (95% CI) | P value | P for interaction |
| --- | --- | --- | --- | --- | --- |
| Age |  |  |  |  | **0.5** |
| ˂ 60 | 38/351 (10.8) | 8/62 (12.9) | 1.00 (0.46-2.16) | 0.995 |  |
| ≥ 60 | 101/576 (17.5) | 9/67 (13.4) | 0.69 (0.35-1.37) | 0.29 |  |
| Gender |  |  |  |  | **0.021** |
| Female | 73/511 (14.3) | 7/88 (8.0) | 0.46 (0.21-1.00) | 0.051 |  |
| Male | 66/416 (15.9) | 10/41 (24.4) | 1.47 (0.75-2.86) | 0.257 |  |
| Race |  |  |  |  | **0.056** |
| White | 50/529 (9.5) | 5/80 (6.2) | 0.57 (0.23-1.44) | 0.236 |  |
| Asian | 9/38 (23.7) | 0/5 (0.0) | 0.00 (0.00-Inf) | 0.999 |  |
| Black | 8/64 (12.5) | 0/10 (0.0) | 0.00 (0.00-Inf) | 0.998 |  |
| Other | 72/296 (24.3) | 12/34 (35.3) | 1.30 (0.70-2.40) | 0.41 |  |
| Hypertension |  |  |  |  | **0.333** |
| No | 82/460 (17.8) | 8/61 (13.1) | 0.60 (0.29-1.24) | 0.167 |  |
| Yes | 57/467 (12.2) | 9/68 (13.2) | 1.01 (0.50-2.05) | 0.972 |  |
| Diabetes |  |  |  |  | **0.775** |
| No | 109/729 (15.0) | 15/111 (13.5) | 0.80 (0.47-1.38) | 0.427 |  |
| Yes | 30/198 (15.2) | 2/18 (11.1) | 0.66 (0.16-2.78) | 0.574 |  |
| Heart failure |  |  |  |  | **0.781** |
| No | 120/855 (14.0) | 16/126 (12.7) | 0.80 (0.47-1.35) | 0.403 |  |
| Yes | 19/72 (26.4) | 1/3 (33.3) | 1.06 (0.14-7.99) | 0.957 |  |
| Coiling |  |  |  |  | **0.934** |
| No | 120/777 (15.4) | 13/95 (13.7) | 0.81 (0.45-1.43) | 0.463 |  |
| Yes | 19/150 (12.7) | 4/34 (11.8) | 0.84 (0.28-2.50) | 0.752 |  |
| Sepsis |  |  |  |  | **0.558** |
| No | 41/486 (8.4) | 3/60 (5.0) | 0.60 (0.18-1.93) | 0.389 |  |
| Yes | 98/441 (22.2) | 14/69 (20.3) | 0.82 (0.47-1.44) | 0.494 |  |
| SAPS Ⅱ |  |  |  |  | **0.98** |
| ˂ 45 | 91/821 (11.1) | 11/113 (9.7) | 0.75 (0.40-1.41) | 0.375 |  |
| ≥ 45 | 48/106 (45.3) | 6/16 (37.5) | 0.75 (0.32-1.76) | 0.511 |  |
| GCS |  |  |  |  | **0.343** |
| ˂ 9 | 31/124 (25.0) | 3/19 (15.8) | 0.52 (0.16-1.71) | 0.284 |  |
| ≥ 9 | 108/803 (13.4) | 14/110 (12.7) | 0.86 (0.49-1.50) | 0.599 |  |
| no. of events / total no. (%) | | | | | |

S6 Table. Sensitivity analysis: subgroup analysis of PRR threshold [Hospital mortality]

| Subgroup | < 22.6 | ≥ 22.6 | HR (95% CI) | P value | P for interaction |
| --- | --- | --- | --- | --- | --- |
| Age |  |  |  |  | **0.206** |
| ˂ 60 | 41/351 (11.7) | 9/62 (14.5) | 1.20 (0.58-2.46) | 0.628 |  |
| ≥ 60 | 135/576 (23.4) | 11/67 (16.4) | 0.66 (0.36-1.23) | 0.191 |  |
| Gender |  |  |  |  | **0.033** |
| Female | 86/511 (16.8) | 9/88 (10.2) | 0.53 (0.27-1.05) | 0.068 |  |
| Male | 90/416 (21.6) | 11/41 (26.8) | 1.43 (0.76-2.68) | 0.269 |  |
| Race |  |  |  |  | **0.021** |
| White | 73/529 (13.8) | 7/80 (8.8) | 0.56 (0.26-1.23) | 0.15 |  |
| Asian | 9/38 (23.7) | 0/5 (0.0) | 0.00 (0.00-Inf) | 0.998 |  |
| Black | 11/64 (17.2) | 0/10 (0.0) | 0.00 (0.00-Inf) | 0.998 |  |
| Other | 83/296 (28.0) | 13/34 (38.2) | 1.50 (0.83-2.69) | 0.177 |  |
| Hypertension |  |  |  |  | **0.467** |
| No | 97/460 (21.1) | 9/61 (14.8) | 0.67 (0.34-1.32) | 0.246 |  |
| Yes | 79/467 (16.9) | 11/68 (16.2) | 0.93 (0.49-1.75) | 0.818 |  |
| Diabetes |  |  |  |  | **0.875** |
| No | 132/729 (18.1) | 17/111 (15.3) | 0.81 (0.49-1.35) | 0.418 |  |
| Yes | 44/198 (22.2) | 3/18 (16.7) | 0.75 (0.23-2.42) | 0.63 |  |
| Heart failure |  |  |  |  | **0.764** |
| No | 153/855 (17.9) | 19/126 (15.1) | 0.80 (0.50-1.30) | 0.371 |  |
| Yes | 23/72 (31.9) | 1/3 (33.3) | 1.13 (0.15-8.45) | 0.903 |  |
| Coiling |  |  |  |  | **0.557** |
| No | 155/777 (19.9) | 15/95 (15.8) | 0.77 (0.45-1.30) | 0.325 |  |
| Yes | 21/150 (14.0) | 5/34 (14.7) | 1.07 (0.40-2.85) | 0.886 |  |
| Sepsis |  |  |  |  | **0.191** |
| No | 55/486 (11.3) | 3/60 (5.0) | 0.43 (0.13-1.38) | 0.156 |  |
| Yes | 121/441 (27.4) | 17/69 (24.6) | 0.93 (0.56-1.55) | 0.779 |  |
| SAPS Ⅱ |  |  |  |  | **0.578** |
| ˂ 45 | 123/821 (15.0) | 13/113 (11.5) | 0.72 (0.41-1.28) | 0.268 |  |
| ≥ 45 | 53/106 (50.0) | 7/16 (43.8) | 0.92 (0.42-2.04) | 0.846 |  |
| GCS |  |  |  |  | **0.63** |
| ˂ 9 | 36/124 (29.0) | 4/19 (21.1) | 0.63 (0.22-1.78) | 0.382 |  |
| ≥ 9 | 140/803 (17.4) | 16/110 (14.5) | 0.82 (0.49-1.38) | 0.457 |  |
| no. of events / total no. (%) | | | | | |

5. Address Clinical Implications: Acknowledge that PRR-guided interventions (e.g., platelet transfusion) are not validated and require prospective trials. Recommend future studies to evaluate PRR dynamics and combined platelet/RBC therapies.

**Response:** We really appreciate your careful examination and question. In the discussion part, we clearly pointed out that the intervention measures based on PRR (such as platelet transfusion) have not been fully verified, and prospective clinical trials are needed to further evaluate their safety and effectiveness. In the last paragraph, we also recommend future studies to evaluate PRR dynamics and combined platelet /RBC therapies.

Page 15 Line 416-421: “Because the dynamic change information of PRR can not be obtained, there may be some limitations in clinical practice, such as whether to carry out platelet transfusion and when to carry out treatment. Future prospective trials should evaluate whether PRR-guided interventions (e.g., early platelet transfusion for PRR<22.6) improve outcomes.”

Page 15 Line 421-424: “For example, the specific location of aneurysm characteristics (location, size and shape) and the potential impact of intervention timing (treatment at different times after bleeding) on the research results. ”

6. Editorial Improvements: Correct inconsistencies in notation (e.g., "Q4 (≥19.42)" vs. "Q4 (219.42)" in Results). Ensure clarity in statistical model descriptions (e.g., Model II adjustments).

**Response:** We thank the reviewer for the careful reminder. We carefully checked the full manuscript to ensure the consistency of symbol use, and made unified amendments to inconsistent symbols such as "Q4 (≥19.42)" and "Q4 (219.42)".

We sincerely appreciate the time and effort that you have invested in evaluating our manuscript. We hope that the correction will meet with approval. Once again, thank you very much for your comments and suggestions.
